# An Account of the Extraction of an Extraneous Substance from the Rectum

**Published:** 1795

**Authors:** William Blair

**Affiliations:** Surgeon of the Lock Hospital; and of the General Dispensary in Newman Street, St. Mary-Le-Bone.


### VI. *An Account of the Extraction of an extraneous Substance from the Rectum. By Mr.* William Blair, *Surgeon of the Lock Hospital; and of the General Dispensary in Newman Street, St. Mary-le-bone.*

ON Tuesday, the 25th of March last, a French gentleman was sent to me by an Apothecary in this neighbourhood, complaining of a pungent, hot, and irritating sensation in the *rectum*; which was considerably augmented during every evacuation *per anum*. These painful symptoms had commenced on the preceding Sunday, and continued to encrease in so alarming a manner, that, upon the day following, he was induced to examine with his finger, whether or not any foreign substance, or other cause of his uneasiness, could be discovered in the intestine. He had the good fortune to feel something in the rectum, which he thought was unnatural, but could not remove it; and therefore he applied the next day for chirurgical assistance.

Having submitted the patient to a proper examination, I readily perceived an hard body confined in the interior membrane of the intestine. With the help of a pair of forceps, I extracted extracted two portions of a brittle black ſubſtance; which, on careful inſpection, appeared to be bread toaſted nearly to a cinder: the two pieces, which were whole before the extraction was attempted, might be together about an inch in length, half an inch in width, and one third of an inch in diameter.

The patient remembered to have ſwallowed ſomething with conſiderable difficulty two days before, while partaking of ſome ſoup; which was probably the ſame morſel of bread that diſtreſſed him upon this occaſion.

Does it not appear from this caſe, that bread when toaſted is leſs fit for digeſtion than ſome perſons would have us believe; and that it affords but little nouriſhment compared with that which is moderately baked?

However trifling the circumſtances of the above caſe may be regarded in its earlieſt ſtage; there can be no doubt entertained of the probability of its terminating very ſeriouſly, if the patient had not applied for ſpeedy relief: inflammation, abſceſs, and all their conſequences, might have enſued, if the efforts of nature, or the power of aperient and antiphlogiſtic remedies had alone been truſted to.

In ſimilar inſtances, without loſing time by endeavours endeavours to relieve the patient's ſufferings by medicine, it will be immediately proper to ſubject him to a careful examination. If the ſimple introduction of a finger be inſufficient to diſengage the extraneous body, and it can be felt adhering to the *rugæ*, or piercing the coats of the rectum, a pair of blunt-pointed ſciſſars, or forceps, (as the caſe may indicate) ſhould be gently conducted upon the finger, in order to divide, break in pieces, or looſen the foreign ſubſtance: if a pointed bone, or other hard and ſharp body, ſhould be confined acroſs the gut, endangering the neighbouring parts, it will be prudent to empty the urinary bladder, previous to any attempt to remove it by mechanical means: and, ſhould the pain, and other ill effects become urgent, it might be neceſſary, after milder methods had proved ineffectual, to make a judicious inciſion either into the rectum, or circumjacent integuments, as the peculiarities of the caſe ſhould require to facilitate the extraction. To obviate the inflammation, and its concomitant ſymptoms, leeches, anodyne and laxative clyſters, with the uſual antiphlogiſtic remedies, ought to be diligently employed.

Inſtances of the kind above related, with

ſuitable remarks, are recorded by ſeveral practical authors; but the reader may ſpare himſelf the trouble of peruſing ſome of them, by conſulting the *Memoires de l'Académie Royale de Chirurgie*, Tom. I. p. 540, et ſeq. 4to Edit.

*Newman Street, Oct.* 6, 1794.

